# The infinite game in the public healthcare system: don’t stop playing

**DOI:** 10.1007/s44250-022-00007-5

**Published:** 2022-11-21

**Authors:** Vanni Agnoletti, Rodolfo Catena, Francesca Bravi, Maria Teresa Montella, Luca Ansaloni, Emanuele Russo, Fausto Catena

**Affiliations:** 1 Department of Surgery and Trauma. Anesthesia and Intensive Care Unit, Maurizio Bufalini Hospital, Viale Giovanni Ghirotti, 286, Zipcode 47521 Cesena, Italy; 2 Azienda Unità Sanitaria Locale Della Romagna, Romagna, Italy; 3 Global Business School for Health, UCL, London, UK; 4 Local Healthcare Authority of Romagna, Ravenna, Italy; 5 Outcome Research, Healthcare Administration, IRCCS Istituto Romagnolo Per Lo Studio Dei Tumori (IRST) “Dino Amadori”, Forlì, Italy; 6 Department of Clinical, Diagnostic and Pediatric Sciences, University of Pavia, Pavia, Italy; 7 IRCCS Policlinico San Matteo Foundation, General Surgery, Pavia, Italy; 8 Department of Surgery and Trauma. Acute Care Surgery Unit, Maurizio Bufalini Hospital, Cesena, Italy

**Keywords:** Game, Finite, Infinite, Healthcare system

In James P Carse’s 1986 book, *Finite and Infinite Games: a vision of life as play and possibility* [1], the author defined two kinds of game: finite and infinite, with different characteristics. A finite game is played for the purpose of winning; an infinite game is played for the purpose of continuing the play [2].

The finite game has a winner and a loser (all players agree on this), with rules of play, a beginning, a middle, and an end, and a designated place where it is played. The infinite game has no winner and loser; it never ends, and there are rules that necessarily have to change during play. Rules have the purpose of preventing anyone from winning, and of bringing as many people as possible into the play.

Finite players play within boundaries, and infinite players play with boundaries, which they use as part of the game. A volleyball team, for example, plays on a court that is divided into equal square halves by a net. The court is defined by lines, and the lines are the boundaries; each team tries to score points by landing a ball on the other team’s court. Actors and actresses instead play with boundaries; they can act on a stage or they can get down from it and act close to the audience, playing with the boundaries.

Date, place, and membership of a finite game are externally defined while they are internally defined for an infinite game [2]. The Champions Football League final, for example, is played with externally defined rules, and teams cannot change them because it is an official game. In comparison, amateur football teams can play with internally defined rules like score (e.g. the game ends after the first goal), length of time, and the number of possible players that can be substituted. Ideally, finite and infinite games have only one thing in common: all players play freely.

In 2019, Simon Sinek applied Carle’s game theory to business in order to develop an infinite mindset for successful organizations [3].

According to Sinek, those seeking to adopt an infinite mindset must follow five practices: (1) advance a “just cause” that defines who you are and where you are going. A just cause is open to all those who would like to contribute; (2) “build trusting teams”. A team is not just a group of people who work together; it is a group of people who trust each other. People who are part of a team should be free to express their concerns; (3) “study worthy rivals”, as their value shows you where you can improve and grow; (4) “prepare for existential flexibility”, which is the capacity to initiate an extreme disruption to a business model or strategic course in order to more effectively advance your cause; (5) “demonstrate the courage to lead” [3].

To succeed in the infinite game of business, people must stop thinking about who wins or who’s the best, and start thinking about how to build organizations that are strong and healthy enough to stay in the game for many generations to come.

So a question arises: can we apply the infinite game to the Healthcare System? The Healthcare System (HS) plays an infinite game because the need for care will last forever. Over years, many proposals of healthcare classification have been made, the most widely used being “base 3”: voluntary insurance (VI), social health insurance (SHI), and national health service (NHS) [4], while others have proposed classifications on “base 4” [4]. Wendt et al. theorized 27 different possible combinations [5]. Rothgang [6] and Mossialos [7] suggested regarding the healthcare system as a triangle created by users, providers, and insurers. In this article, the authors decided to focus on just two systems to bring relevant insight to the discussion: the universalistic healthcare system (UHS) and the private healthcare system (PHS).

The UHS plays an infinite game with the aim of continuing the play without financial benefit, while PHS may play for business; the game may restart every time a new patient enters and money has been earned. PHS plays a finite game.

All systems have the same players such as patients, medical, and non-medical personnel, and infrastructures (hospitals and similar). While patients always play to win the game, it is different for doctors and nurses; some of them play for the purpose of continuing the game (UHS), and others to win (PHS).

Patients are not free to choose to stay in the game, and they cannot change the rules; doctors and nurses freely stay in the game with rules that can and must change. For years it was thought that healthcare rules were difficult or impossible to change (as it is for finite games), but since entering the covid era we have learnt that this is not true. Furthermore, it is possible for patients, doctors, and nurses to change and move from different hospitals and different countries.

Patients could be considered as part of the finite game because their game has a temporal limit (that is to say, it ends), but they are part of an infinite game. The finite game of patients could be considered as part of the infinite game of care, because there will always be patients to take care of, and doctors and nurses too. The healing–recovery process generates winners when patients are healthy again, or losers if they are not.

Nonetheless, infinite games can contain small, finite games within their bigger picture. The healing–recovery process (life and death) is considered a finite game because it cannot be repeated, and obviously doctors and nurses cannot stop the game if they win or if they lose patients (patients lost the game); they have to carry on the infinite game with other patients.

Carse states that the finite game generates a winner who is remembered more than the loser: he/she wins a title [8], which is the acknowledgment for others that they have been the winner of a particular game, and this title makes players visible. Patients in the universalistic or private healthcare systems can win their “healing titles,” while doctors and nurses in UHS cannot because they do not play for victory or for a title. Conversely, in PHS doctors and nurses can have their own title as part of a competitive style of hospital management.

We would like to invite readers to overcome the division between USH and PHS, because it is not only ineffective, but also dangerous to divide patients into winners and losers.

Human beings cannot defeat death, and in this sense we are all losers from the beginning of the game. For this reason, patients should be helped to consider their finite diseases as part of an infinite game. It is not a matter of philosophy, but more a question of how patients as players decide to play their game. They can choose to stop the game or to carry on, and any HS should help them, with their consent, to be part of an infinite game – even if it is obvious they will not succeed.

This way of thinking can affect healthcare culture: as patients, doctors, and nurses we are all part of an infinite game that we play as finite or infinite players. As a patient, unfortunately anyone can participate, at any time during his/her life, in this finite game. As doctors or nurses we should remember that we are part of an infinite game with no limits of time or energy.

Doctors and nurses must continue to play even if it is difficult to have the right motivation. Health personnel should not be induced to look back so far to find the deep reason of their motivation, because doctors and nurses know exactly the world they want to live in: to quote Sinek “they have a clear vision of the future and surely they are open-minded to new contributions.”

Powell et al. described medicine and surgery as chaotic and indeterminate with delusion for employees that is behind the corner [9]. These authors consider this condition as part of the McNamara fallacy in medicine characterized by the risk of ethical fading [9, 10].

Ethical fading is a cultural state that allows people to act in unethical ways in order to advance their own interests, while believing falsely that they have not compromised their own principles [3, 9].

Ethical fading modifies and transforms the infinite game of healing for doctors, nurses, and other healthcare personnel into a finite one. Healthcare players start to play within boundaries represented, for example, by economic issues, such as “there is no money for hospitals,” by the effects of SARS-CoV-19, by wars, by inflation, or simply by individual goals.

Tax revenue, for example, in Italy in 2021 was 555 billion euros, 54 more than 2020 (+ 10.7%) and only 2 billion went to the HS in 2022 (120 billion in 2021 versus 122 billion in 2022). Considering that inflation is 8.4%, we should increase it by 10 billion to stay at the 2021 level; all that in the wake of increases in the cost of food, an energy crisis, and war [11–13].

The UHS is experiencing a lack of resources with serious consequences for the quality of patient care [14], and the professional work environment for all healthcare providers.

Medical education and research are examples of infinite games because they are concrete and unrepeatable, they have changeable rules, and they last forever – with the aim that doctors and nurses will play forever.

The HS, represented by medical personnel, should be obliged to change the rules due to ethical fading into something different, universal, and valuable; they stand on the principle that medicine is ethical, but we should not ignore any fading.

Culture as an infinite game is changing for better or worse, but the “change” is not easy to realize, because our brain is reluctant to change [15]. It demands too much energy to do things for the first time, while repeating an action is less energy consuming.

The HS plays an infinite game every time, for example, additional time is used to change, to improve, to study, to remember other players, to take care of patients, to discuss, and to move on towards a better future.

Within the game and between players lies the idea of trust. Players have to trust each other to follow the rules (finite game) or to change them (infinite game). The game creates a relationship between players even if they do not want that relationship, or they are unaware of it. As doctors and nurses we should aim to be good players; the infinite game is linked to a continuing improvement, and players should invest in themselves to be the best players they can be. D’Angelo et al. sought to explain how to be a good surgeon from Aristotle’s point of view, and a possible answer lies in phronetic knowledge. This is the ability to take the universal epistemic knowledge of the human body, combined with the technical capacity to fix the body, and make decisions on how to do this across complex environments [16]. Phronetic knowledge cannot be taught; it takes time to be a good and wise surgeon, something that a young practitioner needs to acquire. The time of an infinite game. The established relationship between players is complex, and today a realignment is needed between professionals and patients [17–19], professionals and managers, professionals, and policy makers [20], but also between policy makers and citizens.

Changes in the international structure of healthcare professions are needed, and most likely in the structure of the healthcare systems too. Carse wrote that a finite player consumes time, and an infinite one generates it. Surely the correct use of time is mandatory to stay in the game, to be the best players we can be, to gain experience, to teach other players, and to take care of finite players. We must play “within time.”

## Data Availability

Data sharing not applicable to this article as no datasets were generated or analysed during the current study.
